# ﻿*Anormalousliu* sp. nov.: a first record and a new species of the genus *Anormalous* Liu, 2011 (Orthoptera, Tettigoniidae, Phaneropterinae) from India

**DOI:** 10.3897/zookeys.1078.75499

**Published:** 2021-12-16

**Authors:** Muzamil Syed Shah1, Mohd Kamil Usmani1

**Affiliations:** 1 Section of Entomology, Department of Zoology, Aligarh Muslim University, Aligarh 202002, India Aligarh Muslim University Aligarh India

**Keywords:** *Anormalous*, India, Kashmir, new species, Phaneropterinae

## Abstract

The Phaneropterinae, commonly known as the bush katydids, are among the most diverse tettigoniids in the world. A new species *Anormalousliu***sp. nov.** is described from Kashmir, India. This is the second species in the short-winged genus *Anormalous*. It is differentiated from the other species from China by the absence of posterior apical spurs on the fore and mid tibiae, the male subgenital plate with two long cylindrical lobes fused with each other and blunt at the apices, and the male stridulatory area longer than broad. We include a key to species in the genus *Anormalou*. The holotype has been deposited in the Museum of Zoology Department, Aligarh Muslim University, Aligarh Uttar Pradesh, India.

## ﻿Introduction

Katydids show an incredible diversity of forms and species (Heller et al. 2014), with many species reported from India. Some katydids sporadically become very obvious due to a sudden spurt in their population size due to weather conditions (Rentz 2010). Important work on the taxonomy and distribution of the Tettigoniidae (including Phaneropterinae) of India include those of Barman and Srivastava (1976), Shishodia (2000), Barman (2003) and Shishodia et al. (2010). The Phaneropterine occupy a wide range of open habitats (Kocarek and Holusa 2006). Recently, Nagar et al. (2014, 2015) and Farooqi et al. (2021) reported new species of Phaneropterinae from India.

The genus *Anormalous* most resembles the genera in the tribe Ducetiini in the lateral lobe of the pronotum, the tympanum structure, the fore tibiae, and the absence of styli in male subgenital plate, but differs by the particular tegminal structure (Liu 2011). The genus was established for the species *Anormalouszhangi* Liu, 2011 from southern China, with only male specimen reported. The new species described herein can be assigned to the genus *Anormalous* based on similarities of the tegminal structure, but differs in various morphological characters described below.

## ﻿Materials and methods

During a field survey conducted in 2021 at different places in the Kashmir region, the specimens were collected by handpicking or with the help of sweep nets. Out of all collected samples, one male, and three females of the new species were found. They were preserved in alcohol and brought to the laboratory for identification. The specimens were examined under a stereo zoom binocular microscope. Genitalia were observed after cleaning with KOH. Photographic images were done using a DSLR camera with macro-lens. All body parts were measured using a vernier caliper. Both the holotype and paratype have been deposited in the Museum of Zoology Department, Aligarh Muslim University, Aligarh Uttar Pradesh, India.

## ﻿Results and discussion

### ﻿ Anormalou

Taxon classificationAnimaliaOrthopteraTettigoniidae

Liu, 2011

465C8BD2-E35B-5826-AB86-DF16F2F43F76

#### Description.

**Figures 1–4.**
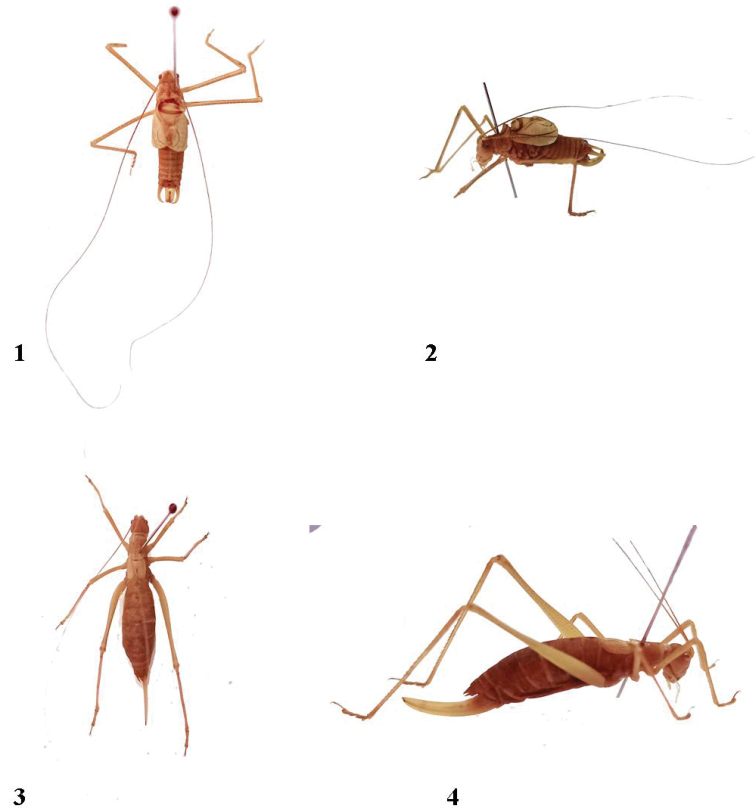
*Anormalousliu* sp. nov. **1–2** Holotype male and **2–3** paratype female.

**Figures 5–13.**
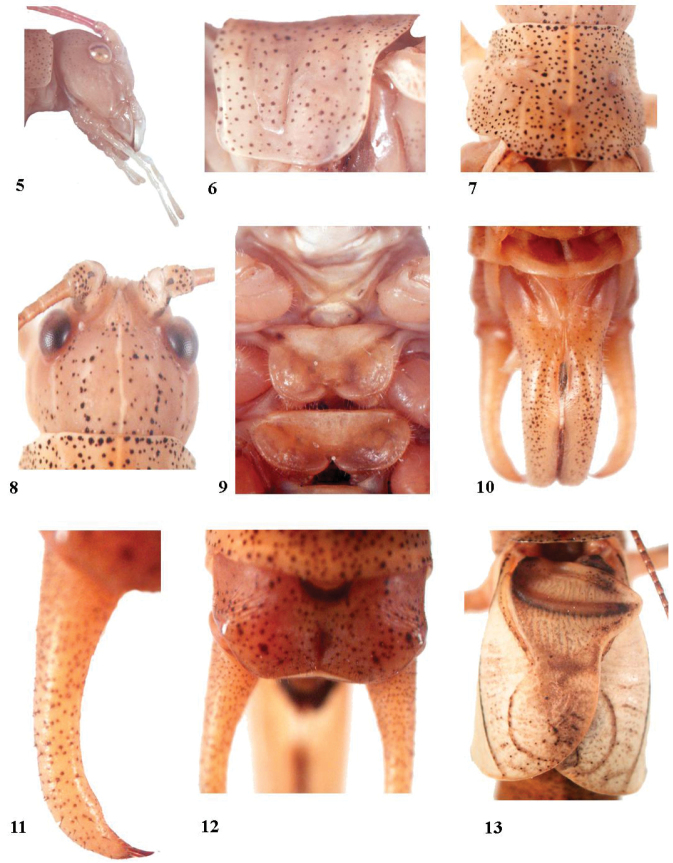
Characters of the holotype male *Anormalousliu* sp. nov. **5** head lateral **6** lateral view of pronotum **7** dorsal view of pronotum **8** fastigium **9** sternum **10** subgenital plate **11** cerci **12** male last tergite **13** tegmen.

Small sized body (Figs 1–4), light green, head more or less oval in shape (Fig. 5), fastigium dorsally sulcate with conical apex, narrower than first antennal segment (Fig. 8). Lateral lobe of pronotum distinctly longer than high (Fig. 6). Pronotal disc with prozona smooth and metazona flat, without lateral carinae (Fig. 7). Lateral lobe of pronotum with shallow humeral sinus. Eyes large and bulging outwards, antenna long (Figs 1–4), male subgenital plate elongate, notch at apical margin present or absent, devoid of distinct styli (Fig. 10), female tegmen comparatively shorter than male’s with visible longitudinal veins (Figs 3, 4), last abdominal tergite rounded (Fig. 16), and ovipositor weekly curved (Fig. 18).

#### Distribution.

China and India (Kashmir)

### ﻿Key to species of the genus *Anormalous* (males only)

**Table:** 

1	Posterior apical spurs on fore and mid tibiae present, male subgenital plate elongate, split from basal third into two triangular lobes, notch narrow triangular, lateral margin of lateral lobe tapering towards apices, male stridulatory area broader than long	***Anormalouszhangi* Liu, 2011**
–	Posterior apical spurs on fore and mid tibea absent, male subgenital plate with two long cylindrical lobes fused with each other, blunt at the apices (Fig. 10), with a small notch at anterior portion, male stridulatory area longer than broad (Fig. 13)	***Anormalousliu* sp. nov.**

### ﻿ Anormalous liu sp. nov

Taxon classificationAnimaliaOrthopteraTettigoniidae

B92E59FB-E825-5B0A-A893-8B5325D05C30

http://zoobank.org/49E3B7CD-3911-4E83-9D04-133C29361A30

Figures 1–4 , 5–13 , 14–21

#### Description.

**Male**: Small sized body, eyes large and bulging outwards, antenna long and flexible, light green, fastigium dorsally sulcate with conical apex, narrower than first antennal segment. Pronotum saddle shaped; lateral lobe of pronotum distinctly longer than high. Pronotal disc with prozona smooth and metazona flat, without lateral carinae. Lateral lobe of pronotum with shallow humeral sinus. Tegmen short not surpassing the abdomen with longitudinal veins well developed, apex rounded; hind wings not well developed and shorter than tegmen. Fore tibia with two rows of 9 evenly- distributed spines ventrally; mid tibia with 12 spines ventrally and 6 dorsally; prosternum unarmed; mesosternum and metasternum with two more or less rounded lobes. Male last abdominal tergite rounded with a shallow depression; cerci long and cylindrical with pointed apex. Male subgenital plate elongated with two long lobes attached together; small notch at anterior end; apical end without distinct styli.

**Female**: Last abdominal tergite rounded without any incision; subgenital plate small, conical; epiproct long and tongue- shaped; cerci small, slender tapering toward the end; ovipositor long and weekly curved, with small teeth at distal end.

#### Remarks.

The new species differs from the only other species, *Anormalouszhangi*Liu (2011), as follows: male subgenital plate with two long cylindrical lobes fused with each other, blunt at the apices (Fig. 10), male stridulatory area longer than broad (Fig. 13), and absence of posterior apical spurs on fore and mid tibiae.

#### Distribution.

India, Kashmir

#### Etymology.

The name of the species is given after Chun-Xiang Liu who described the genus *Anormalous*.

#### Material examined.

***Holotype*: Male.** India: Jammu and Kashmir; Kashmir, Kupwara, (34.5262°N, 74.2546°E), 01 male, 16.08.2021, on grass, collected by Muzamil Syed Shah deposited in Museum of Zoology Department, Aligarh Muslim University, Aligarh Uttar Pradesh, India.

**Figures 14–21.**
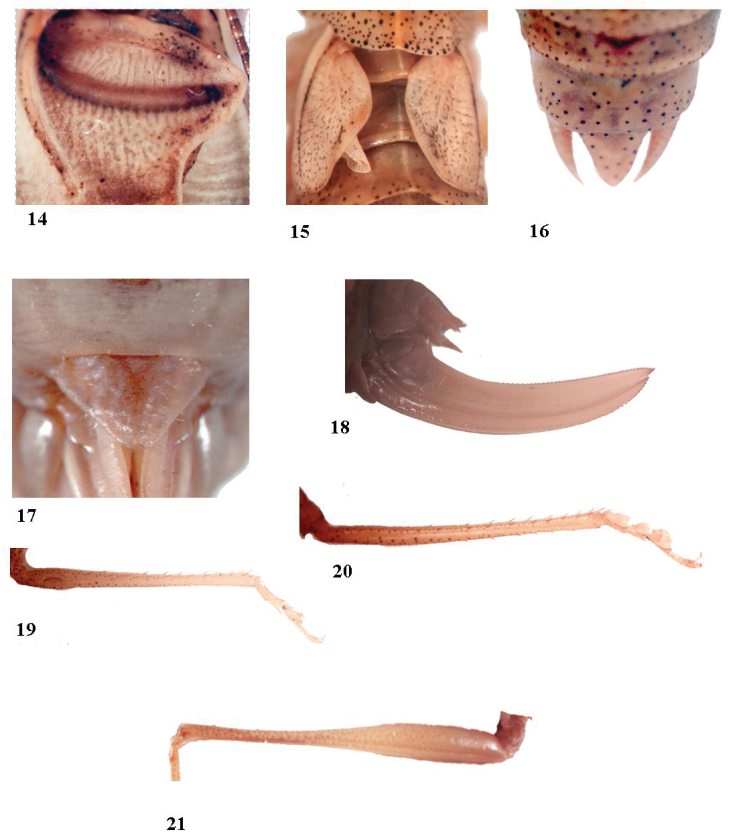
*Anormalousliu* sp. nov. **14** male stridulatory file **15** female tegmen **16** female last tergite **17** female subgenital plate **18** ovipositor **19** fore tibia **20** mid tibia **21** hind femur.

***Paratype*: Female**: India: Jammu and Kashmir; Kashmir, Baramulla, Gulmarg (34.0484°N, 74.3805°E), two females, 20.08.2021, on grass, collected by Muzamil Syed Shah deposited in Museum of Zoology Department, Aligarh Muslim University, Aligarh Uttar Pradesh, India.

## Supplementary Material

XML Treatment for Anormalou

XML Treatment for Anormalous liu

## References

[B1] Barman RS Srivastava GK (1976) On a collection of Tettigoniidae Newsletter 2(3): 93–94.

[B2] Barman RS (2003) Insecta: Orthoptera: Tettigoniidae. Zoological Survey of India, Fauna of Sikkim, State Fauna Series 9(2): 193–201.

[B3] Farooqi MK Ahmed I Usmani MK (2021) A New Species of Genus Ducetia Stal, 1874 (Orthoptera: Tettigonioidea: Tettigoniidae) from India. Transactions of the American Entomological Society 147(1): 1–19. 10.3157/061.147.0102

[B4] Heller KG Hemp C Liu C Volleth M (2014) Taxonomic, bioacoustic and faunistic data on a collection of Tettigonioidea from Eastern Congo (Insecta: Orthoptera). Zootaxa, 3785(3): 343–376. 10.11646/zootaxa.3785.3.2 24872232

[B5] Kocarek P Holusa J (2006) Recent expansion of bush cricket *Phaneropterafalcata* (Orthoptera: Tettigonidae) in northern Moravia and Silesia (Czech Republic). Scrip. Facult. Rer. Nat. Univ. Ostaviensis 163: 207–211.

[B6] Liu CX (2011) Phaneroptera Serville and Anormalous gen. nov.(Orthoptera: Tettigoniidae: Phaneropterinae) from China, with description of two new species. Zootaxa 2979(1): 60–68. 10.11646/zootaxa.2979.1.4

[B7] Nagar R Mal J Swaminathan R (2014) Additions to the reported Elimaea species (Orthoptera: Phaneropteridae: Phaneropterinae) from India. Zootaxa 3860(6): 536–546. 10.11646/zootaxa.3860.6.2 25283289

[B8] Nagar R Mal J Swaminathan R (2015) A note on the new species of the genus Isopsera (Orthoptera: Phaneropteridae: Phaneropterinae) from India. Zootaxa 3964(1): 95–100. 10.11646/zootaxa.3964.1.6 26249423

[B9] Rentz D (2010) A Guide to the Katydids of Australia. CSIRO Publishing, Melbourne, 224 pp. 10.1071/9780643100183

[B10] Shishodia MS Barman RS (2004) Insecta: Orthoptera: Tettigoniidae. Zoological Survey of India, Fauna of Manipur, State Fauna Series 10: 139–145.

[B11] Shishodia MS Chandra K Gupta SK (2010) “An Annotated Checklist of Orthoptera (Insecta) from India. Records of Zoological Survey of India, Occasional Paper No. 314: 283–324.

